# The calcium-sensing receptor: A promising target for prevention of colorectal cancer^☆^

**DOI:** 10.1016/j.bbamcr.2015.02.011

**Published:** 2015-09

**Authors:** Abhishek Aggarwal, Maximilian Prinz-Wohlgenannt, Samawansha Tennakoon, Julia Höbaus, Cedric Boudot, Romuald Mentaverri, Edward M. Brown, Sabina Baumgartner-Parzer, Enikö Kállay

**Affiliations:** a Department of Pathophysiology and Allergy Research, Medical University of Vienna, Vienna, Austria; b INSERM U1088, University of Picardie Jules Verne, Amiens, France; c Division of Endocrinology, Diabetes and Hypertension, Department of Medicine, Brigham and Women's Hospital, Boston, USA; d Department of Internal Medicine III, Medical University of Vienna, Vienna, Austria

**Keywords:** Ca^2 +^, calcium, CRC, colorectal cancer, CaSR, calcium-sensing receptor, PTH, parathyroid hormone, DMEM, Dulbecco's Modified Eagle's Medium, FBS, fetal bovine serum, Zeo, Zeocin, RPLPO, large ribosomal protein, β2M, beta-2-microglobulin, Eef1B2, eukaryotic translation elongation factor 1 beta 2, ANOVA, analysis of variance, CDC, cell division cycle, CDT1, chromatin licensing and DNA replication factor 1, MCM, mini chromosomal maintenance complex, CDX2, caudal type homeobox 2, SI, sucrase isomaltase, BAX, Bcl-2 associated X protein, SCC, Spearman correlation coefficient, EMP, empty, WT, wild type, DN, dominant negative, Calcium-sensing receptor, Tumor suppressor, Colorectal cancer, Colon, Calcium, Calcimimetic, Calcilytic

## Abstract

The inverse correlation between dietary calcium intake and the risk of colorectal cancer (CRC) is well known, but poorly understood. Expression of the calcium-sensing receptor (CaSR), a calcium-binding G protein-coupled receptor is downregulated in CRC leading us to hypothesize that the CaSR has tumor suppressive roles in the colon. The aim of this study was to understand whether restoration of CaSR expression could reduce the malignant phenotype in CRC.

In human colorectal tumors, expression of the CaSR negatively correlated with proliferation markers whereas loss of CaSR correlated with poor tumor differentiation and reduced apoptotic potential. *In vivo*, dearth of CaSR significantly increased expression of proliferation markers and decreased levels of differentiation and apoptotic markers in the colons of *CaSR*/*PTH* double knock-out mice confirming the tumor suppressive functions of CaSR.

In *vitro* CRC cells stably overexpressing wild-type CaSR showed significant reduction in proliferation, as well as increased differentiation and apoptotic potential. The positive allosteric modulator of CaSR, NPS R-568 further enhanced these effects, whereas treatment with the negative allosteric modulator, NPS 2143 inhibited these functions. Interestingly, the dominant-negative mutant (R185Q) was able to abrogate these effects.

Our results demonstrate a critical tumor suppressive role of CaSR in the colon. Restoration of CaSR expression and function is linked to regulation of the balance between proliferation, differentiation, and apoptosis and provides a rationale for novel strategies in CRC therapy.

## Highlights

• The CaSR is a tumor suppressor in the colon inhibiting several hallmarks of cancer.
• Restoration of CaSR expression and function reduces malignant phenotype in CRC.
• Our data provide a rationale to develop CRC therapy stratergies targeting the CaSR.
• Persons with inactivating CaSR mutations could represent a new risk group in CRC.

## 1 Introduction

Garland et al. demonstrated an inverse correlation between dietary calcium (Ca^2 +^) intake and the risk of colorectal cancer (CRC), identifying nutritional Ca^2 +^ as a promising chemopreventive agent [1]. Since then several advances have been made with focus on studies trying to establish risk factors to facilitate suitable intervention in CRC [1–3]. In the colon, Ca^2 +^ exerts its functions through various mechanisms, including binding toxic bile acids and ionized fatty acids, to form insoluble soaps or by regulating cellular proliferation, inducing differentiation and/or stimulating apoptosis [4–12]. However these anti-tumorigenic effects of Ca^2 +^ fail during tumor progression. Although it is clear that Ca^2 +^ exerts chemopreventive features in the colon, the molecular mechanisms by which extracellular Ca^2 +^ modulates cell fate are not fully understood. Finding markers that would allow us to distinguish between an individual's responsiveness to calcium, or a target that could be modulated to restore responsiveness is of utmost importance.

The calcium-sensing receptor (CaSR) has been identified as a key molecule in regulating systemic calcium homeostasis in the parathyroid [13]. The *CaSR* gene encodes a calcium-binding G protein-coupled receptor (GPCR), with an extracellular N-terminal domain (containing the calcium binding sites), joined to the C-terminal domain via a seven transmembrane region (essential for its signaling function). Most orthosteric ligands of the CaSR bind to the large N-terminal domain of the receptor [14]. Several synthetic modulators of the CaSR have been developed in order to modulate CaSR function. Calcimimetic agents like NPS R-568 are positive allosteric modulators of the CaSR, which potentiate the effects of the CaSR by interacting with the 7-transmembrane region of the receptor and inducing conformational changes. Calcilytic agents (e.g. NPS 2143) that are negative allosteric modulators of the receptor act in a similar manner, desensitizing the receptor, and reducing its affinity to its ligands [14,15].

There is strong evidence for the involvement of the CaSR in various functions determining cellular fate [4,16,17]. These functions extend beyond the control of calcium homeostasis; the primary function of the CaSR. It has been suggested that the CaSR is either a tumor suppressor (e.g. in colon and parathyroid) or an oncogene (e.g. in breast and prostate) depending on the site of disease [18]. Expression of colonic CaSR is significantly downregulated during colorectal tumorigenesis [19–21] at least in part, by aberrant DNA methylation and histone deacetylation [19,22]. CRC cells that lack the CaSR have a highly malignant phenotype [23]. Taken together, the epidemiological observation of the inverse relationship between Ca^2 +^ intake and risk of CRC, the observation that CaSR mRNA and protein expression is reduced in human colon tumors, and the observations that CRC cells lacking the CaSR have a malignant phenotype lead to the hypothesis that the CaSR is a tumor suppressor in the colon. However, there are only limited or no data in support of a causal relationship between CaSR expression and de-differentiation and carcinogenesis.

In this study we present evidence that the CaSR is a tumor suppressor in the colon using three distinct approaches: *ex vivo*, *in vivo* and *in vitro*. These models have allowed us to investigate the role of the receptor in the colon and to understand whether restoration of CaSR function can prevent or attenuate the malignant phenotype in CRC.

## 2 Materials and methods

### 2.1 Human patient samples

Fresh frozen tumor tissue and adjacent non-tumor tissue from 54 CRC patients were obtained after written consent. Samples were collected from the General Hospital of Vienna and Rudolfstiftung Hospital in Vienna and snap frozen in liquid nitrogen. A pathologist graded and classified the samples according to the TNM system. Approval by the local ethics committees was obtained prior to the start of the study. Clinicopathological characteristics of the patient cohort are shown in Table 1.

### 2.2 Animals

Mice heterozygous for *CaSR*^ΔExon5^ and *PTH* were bred to generate *CaSR*^+/+^/*PTH*^−/−^ and *CaSR*^−/−^/*PTH*^−/−^ mice as previously described [24]. Ethics approval was obtained from the Institutional Animal Care and Use Committee at Harvard Medical School. Mice were fed standard mouse chow (Harlan Teklad-TD99224, Harlan-Teklad, USA). Age- and sex-matched animals (n = 9/genotype) were sacrificed, the colons were washed in ice cold PBS and stored in RNAlater (Life Technologies, Austria) until RNA extraction was carried out. For the analysis, 1–2 cm of colonic tissue 0.5 cm distal from cecum was used.

### 2.3 RNA isolation, reverse transcription and quantitative-reverse transcription PCR (qRT-PCR)

Total RNA was isolated using TRIzol (Life Technologies) according to the manufacturer's instructions. RNA integrity was checked by agarose gel electrophoresis, and total RNA was reverse transcribed as previously described [25]. qRT-PCR was performed using POWER SYBR GREEN Mastermix (Life Technologies) on a Step One Plus qRT-PCR machine (Life Technologies). Where possible, primers were designed to bridge an exon–exon junction to prevent genomic DNA from being amplified. The ΔΔC_t_ method was used to calculate fold changes in gene expression, relative to housekeeping genes (human beta-actin (hβ-ACTIN) for human patient samples; hβ-ACTIN, human large ribosomal protein (hRPLPO) and human beta-2-microglobulin (hβ2M) for human cell lines; mβ-Actin and mouse eukaryotic translation elongation factor 1 beta 2 (mEef1B2) for mouse colon samples) and normalized to a commercially available total RNA calibrator (Clontech, USA). Primer sequences are described in Supplementary Table S1. Primer sequences for hCDC6, hMCM2, hβ-ACTIN, hRPLPO and hβ2M have been previously described [19,25].

### 2.4 Cell culture

The human CRC cell lines Caco2-15 and HT29 were used in this study. HT29 cells were obtained from American Type Culture Collection (ATCC, USA). The Caco2-15 cells are a sub-clone of Caco2 cells [26] and were kindly provided by Prof. A Quaroni (Cornell University, USA). Cells were regularly maintained in Dulbecco's Modified Eagle's Medium (DMEM) containing 10% fetal bovine serum (FBS) as previously described [19]. Ca^2 +^ concentration in the medium was 1.8 mM, unless otherwise stated. Cells were routinely tested for mycoplasma contamination using the VenorGem Mycoplasma Detection Kit (Minerva Biolabs, Germany) and were periodically authenticated by STR DNA profiling (DNA Diagnostic Center, UK).

### 2.5 Cloning of the human CaSR cDNA

pcDNA3.1 expression vector encoding a full length wild-type CaSR (CaSR-WT) was kindly provided by Dr. AL Magno and Prof. BK Ward (University of Western Australia, Nedlands). hCaSR cDNA was sub-cloned into the HindIII and XbaI restriction enzyme sites of the pcDNA™3.1/Zeo^(+)^ expression vector (Life Technologies). The hCaSR sequence was FLAG-tagged in its C-terminal region using a reverse primer designed to contain, in the 5′ to 3′ direction, the XbaI recognition sequence, the wild-type stop codon sequence, the sequence encoding the FLAG peptide (GACTACAAGGACGACGATGACAAG), and the hCaSR sequence adjacent to the wild-type stop codon. Another set of primers was then used to generate the R185Q dominant negative mutant of the hCaSR (CaSR-DN), using the FLAG tagged hCaSR as a template. After PCR amplification, appropriately digested PCR products were cloned into HindIII/XbaI digested pcDNA™3.1/Zeo^(+)^. Sequence fidelity of the PCR products was checked by sequence analysis.

### 2.6 Cloning and stable transfection of plasmids in Caco2-15 and HT29 cells

Plasmid constructs were cloned using either One Shot^®^ MAX Efficiency^®^ DH5α™-T1^R^ Competent *E. coli* Cells or Topo TA Cloning Kit and electro-competent *E. coli* bacteria following the manufacturer's protocol. Midipreps were performed with PureLink^®^ HiPure Plasmid Midiprep Kit (all Life Technologies).

HT29 and Caco2-15 cells were cultured in 24-well plates to 50–60% confluency. Non-linearized plasmid midipreps were transfected using Lipofectamine™ LTX (Life Technologies) [Caco2-15 (500 ng/well plasmid, 1.25 μl Lipofectamine™ LTX) and HT29 (750 ng/well plasmid, 2.5 μl Lipofectamine™ LTX)] for 48 h. After transfection, cells were cultured in the presence of Zeocin (Caco2-15: 75 μg/ml and HT29: 150 μg/ml) for over six months to select stably transfected cells.

### 2.7 Sequencing DNA from stably transfected Caco2-15 and HT29 cells

DNA was extracted using the DNeasy Blood and Tissue Kit (Qiagen, Germany) according to the manufacturer's instructions. Standard PCR was used to amplify exon 4 of *CaSR* gene using *Taq* polymerase: initial denaturation at 95 °C for 2 min, 30 cycles of denaturation at 97 °C for 30 s, annealing at 65 °C for 60 s, extension at 72 °C for 90 s followed by elongation at 72 °C for 7 min and termination at 4 °C. PCR products were sequenced using the Genetic Analyzer 3130xI (Life Technologies). Primers used were GGCTCTTCTACATTCAG (Fwd) and GAATTCCCGGAAGCCTGGGATCTGC (Rev).

### 2.8 Immunostaining

Cells were stained to detect CaSR expression using a monoclonal antibody against the ADD region in the N-terminal domain of the CaSR sequence. Cells grown on glass cover slips were fixed in 3.7% paraformaldehyde for 20 min, permeabilized with 0.2% Triton-X for 20 min, and blocked with 5% goat serum for 30 min. Cells were incubated with anti-CaSR antibody (1:200, Abcam, UK) for 1 h at room temperature. After extensive washing, samples were incubated with Alexa Fluor 647 goat-anti-mouse antibody (1:1000, Life Technologies). Nuclei were stained with DAPI (1:3000, Roche, Switzerland). Whole slide images were acquired using TissueFAXS (TissueGnostics, Austria). Isotype-specific IgG was used as negative control and human kidney tissue was stained as positive control.

### 2.9 Growth curve assay

Cells were plated in triplicates (initial seeding density: Caco2-15: 5000 cells/cm^2^; HT29: 7500 cells/cm^2^) in 96 well plates in regular growth medium. Cells were harvested after 24, 48 and 72 h in culture. To overcome the possible differences in plating efficiencies between genotypes, data were normalized to the number of viable cells 24 h post-seeding. Doubling time was calculated using the web tool at www.doubling-time.com/compute.php. The assay was performed at least in three independent experiments.

### 2.10 Functional assays

Cells were cultured in the normal growth medium. One week post-confluence, medium was changed to serum-free DMEM supplemented with 5 mg/ml insulin, 5 mg/ml transferrin, and 5 ng/ml sodium selenite (ITS, Life Technologies) as previously described [4]. Cells were treated with the allosteric modulators of CaSR [NPS R-568 (1 μM in DMSO) or NPS 2143 (1 μM in DMSO)] for 48 h. Medium contained 1.8 mM Ca^2 +^ unless otherwise stated. Vehicle-treated cells were used as controls in all experiments.

Proliferation was measured by counting viable cells as previously described [19] with the T10 automated cell counter (BioRad, USA). Differentiation was determined using the Alkaline Phosphatase (ALP) Colorimetric Assay Kit (Abcam). ALP activity per 10^5^ cells was calculated and data presented in units/ml. In HT29 cells, differentiation was measured using qRT-PCR to measure mRNA expression of the differentiation markers, sucrase isomaltase and caudal type homeobox 2 (CDX2). Apoptosis was evaluated using the Apo-ONE Homogeneous Caspase 3/7 Assay Kit (Promega, Germany), and data were normalized to the number of viable cells. All assays were performed according to the manufacturer's instructions and repeated at least in three independent experiments.

### 2.11 Statistical analysis

Paired t-tests were computed to compare the expression of genes between tumor and adjacent non-tumor samples of the same patient. Correlation analysis was performed using the nonparametric Spearman correlation test. For group comparison, Analysis of Variance (ANOVA) tests were performed followed by Tukey's post-test. Non-normally distributed data were log-transformed to achieve normal distribution. p values < 0.05 were considered statistically significant. SPSS (IBM, USA) was used to perform all statistical calculations and graphs were plotted using GraphPad Prism (GraphPad Software Inc., USA).

## 3 Results

### 3.1 CaSR mRNA expression positively correlates with the expression of differentiation and pro-apoptotic markers, and negatively correlates with proliferation markers in CRC patients

**Fig. 1:**
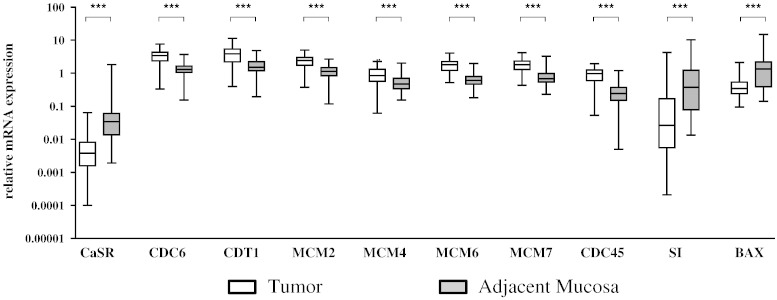
Expression of the CaSR and markers of neoplastic transformation in colorectal tumors. Tumor samples (mainly grade 2) and adjacent mucosa from 54 CRC patients were investigated for gene expression of the CaSR, the proliferation markers CDC6, CDT1, MCM2, MCM4, MCM6, MCM7, and CDC45, the differentiation marker SI and the pro-apoptotic marker BAX. Median, interquartile range and whiskers representing 5th to 95th percentile are shown. Statistical significance was calculated using paired t-tests. ***p < 0.001.

Expression of the CaSR is significantly down-regulated in CRC, possibly impairing the anti-tumorigenic effects of Ca^2 +^. To predict if loss of CaSR in CRC is linked to tumorigenesis, we performed correlation analyses between CaSR expression and genes regulating neoplastic transformation in tumor samples (mainly grade 2) from 54 CRC patients (Table 1), compared with samples of the adjacent non-tumorous mucosa from the same patient. We examined the mRNA expression of a ‘DNA replication signature’ that has been previously reported to be deregulated in transformed colon [27]. DNA replication factors cell division cycle 6 (CDC6), chromatin licensing and DNA replication factor 1 (CDT1), mini chromosomal maintenance complex (MCM2, MCM4, MCM6, MCM7) and CDC45 were all significantly up-regulated in tumor samples compared with the adjacent mucosa of the same patient (p < 0.001, Fig. 1). Spearman correlation analyses showed significant negative correlation between CaSR expression and the expression of the DNA replication genes (p < 0.001, Table 2). As > 90% of our patient cohort (51 out of 54, Table 1) were of grade 2, and we had only one stage IV patient with distant metastasis, we were unable to assess a correlation between CaSR expression and tumor grade or stage.

The expression of the differentiation marker, sucrase isomaltase (SI) and the pro-apoptotic marker, Bcl-2 associated X protein (BAX) were significantly down-regulated in the tumor samples compared with the adjacent mucosa (n = 54, p < 0.001, Fig. 1). Spearman correlation analyses showed significant, positive correlation between CaSR expression and the expression of SI and BAX (p < 0.001, Table 2).

### 3.2 CaSR regulates transcription of proliferation, differentiation and apoptosis-related genes *in vivo*

**Fig. 2:**
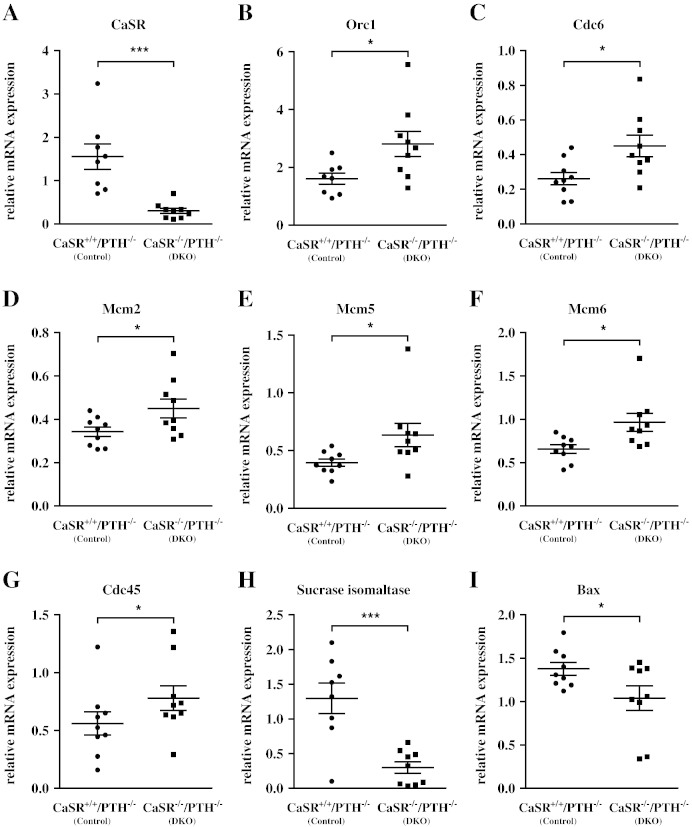
Effect of colonic CaSR on regulation of genes modulating proliferation, differentiation, and apoptosis *in vivo*. Colons sampled from *CaSR*^−/−^/*PTH*^−/−^ (DKO) and *CaSR*^+/+^/*PTH*^−/−^ (control) mice were investigated for gene expression. mRNA expression of CaSR (A). mRNA expression of the proliferation markers Orc1 (B), Cdc6 (C), Mcm2 (D), Mcm5 (E), Mcm6 (F) and Cdc45 (G). mRNA expression of differentiation marker, Si (H), and the pro-apoptotic marker, Bax (I). Individual data points are shown. Bars represent median ± SEM. Statistical significance was calculated using unpaired t-tests. n = 9 animals/group, *p < 0.05, ***p < 0.001.

We investigated the consequence of CaSR knock down in a mouse model in which exon-5 of the *CaSR* gene is globally ablated on a PTH-null background to prevent the development of catastrophic primary hyperparathyroidism. qRT-PCR using primers targeting the junction of exons 4 and 5 of CaSR confirmed lack of CaSR mRNA expression in the colons of *CaSR*^−/−^/*PTH*^−/−^ (DKO) mice compared with *CaSR*^+/+^/*PTH*^−/−^ (control) mice (n = 9 animals/genotype, Fig. 2A).

The mRNA expression of genes of the ‘DNA replication signature’ (origin recognition complex 1 (Orc1), Cdc6, Cdc45, Mcm2, Mcm5, and Mcm6) was significantly upregulated in mice lacking the CaSR (DKO, Fig. 2B–G). In contrast, the differentiation marker sucrase isomaltase (Fig. 2H), and the pro-apoptotic marker, Bax (Fig. 2I) were significantly downregulated in the colons of these DKO mice.

Taken together, the data from the *ex vivo* and the *in vivo* study show that colonic cells with reduced CaSR expression have a significant derangement in molecular markers of neoplastic progression.

### 3.3 Stable transfection of Caco2-15 and HT29 cells

We studied the effect of CaSR on hallmarks of cancer in two CRC cell lines: the Caco2-15 cells representative of differentiated tumors, and the highly malignant HT29 cells representative of undifferentiated tumors [26,28,29]. Endogenous CaSR expression is negligible in HT29 cells but detectable in Caco2-15 cells. We stably transfected the Caco2-15 and HT29 cells with plasmids containing either a full-length wild-type CaSR (CaSR-WT), or a functionally inactive dominant negative mutant (R185Q, CaSR-DN). Cells transfected with the empty pcDNA™3.1/Zeo^(+)^ plasmid (EMP) served as controls.

**Fig. 3:**
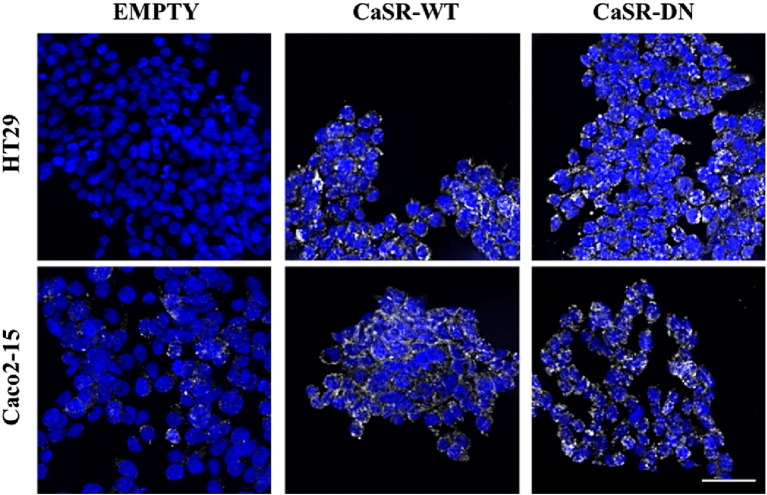
Confirmation of transfection in CRC cell lines. Expression of CaSR protein as determined by immunofluorescence. White bar represents 50 μm.

Sequence fidelity of the plasmids was confirmed by sequence analysis. DNA sequencing of exon 4 of the *CaSR* gene confirmed successful integration of transfected vectors. CaSR-DN cells expressed the R185Q mutation, whereas CaSR-WT cells expressed the wild-type sequence (Gene Id: 846). PCR using primers directed against the region encompassing the FLAG peptide and exon 7 to exclude amplification of endogenous CaSR confirmed transfections of all 3 constructs in both cell lines (data not shown). Immunostaining using anti-CaSR antibody showed basal CaSR protein in Caco2-15^EMP^ cells whereas HT29^EMP^ cells did not exhibit any CaSR immuno-reactivity. Upregulation of CaSR protein was seen in cells overexpressing CaSR-WT and CaSR-DN constructs, in both cell lines (Fig. 3).

### 3.4 CaSR modulates growth kinetics of colon cancer cells

To assess the effects of the expression of the various constructs, stably transfected cells were seeded in normal growth medium and viable cells were counted 48 and 72 h post-seeding.

**Fig. 4:**
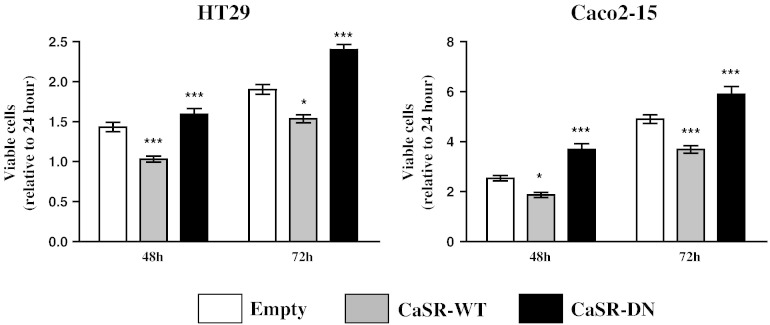
CaSR regulates growth kinetics of HT29 and Caco2-15 cells. Stably transfected cells were counted 24, 48 and 72 h post-seeding. Stable over-expression of CaSR-WT was associated with reduced proliferation rates. Overexpression of CaSR-DN however, was associated with increased growth rates. Cells stably transfected with the empty vector (EMP) were used as controls. Data were normalized to number of viable cells 24 h post-seeding. Bars represent means ± SEM of 3–5 independent experiments. Statistical significance was determined by ANOVA followed by Tukey's post-test. Asterisks above bars indicate significant changes within groups (compared with EMP cells), *p < 0.05, ***p < 0.001.

Both, HT29 and Caco2-15 cells overexpressing the CaSR-WT construct grew significantly slower than control cells transfected with the empty vector, whereas cells expressing the CaSR-DN grew significantly faster (p < 0.001, Fig. 4). Doubling time analysis was consistent with these results (Table 3).

### 3.5 Over-expression of CaSR inhibits proliferation and promotes apoptosis and spontaneous differentiation in Caco2-15 cells

Ca^2 +^ has been shown to have anti-neoplastic effects in the colon by modulating anti-proliferative, pro-differentiating and pro-apoptotic mechanisms. To further understand how the CaSR levels and function influence the anti-tumorigenic properties of Ca^2 +^, stably transfected Caco2-15 cells were treated with either the calcimimetic NPS R-568, or the calcilytic NPS 2143 for 48 h to study how these specific CaSR modulators affect proliferation, spontaneous differentiation and apoptosis.

**Fig. 5:**
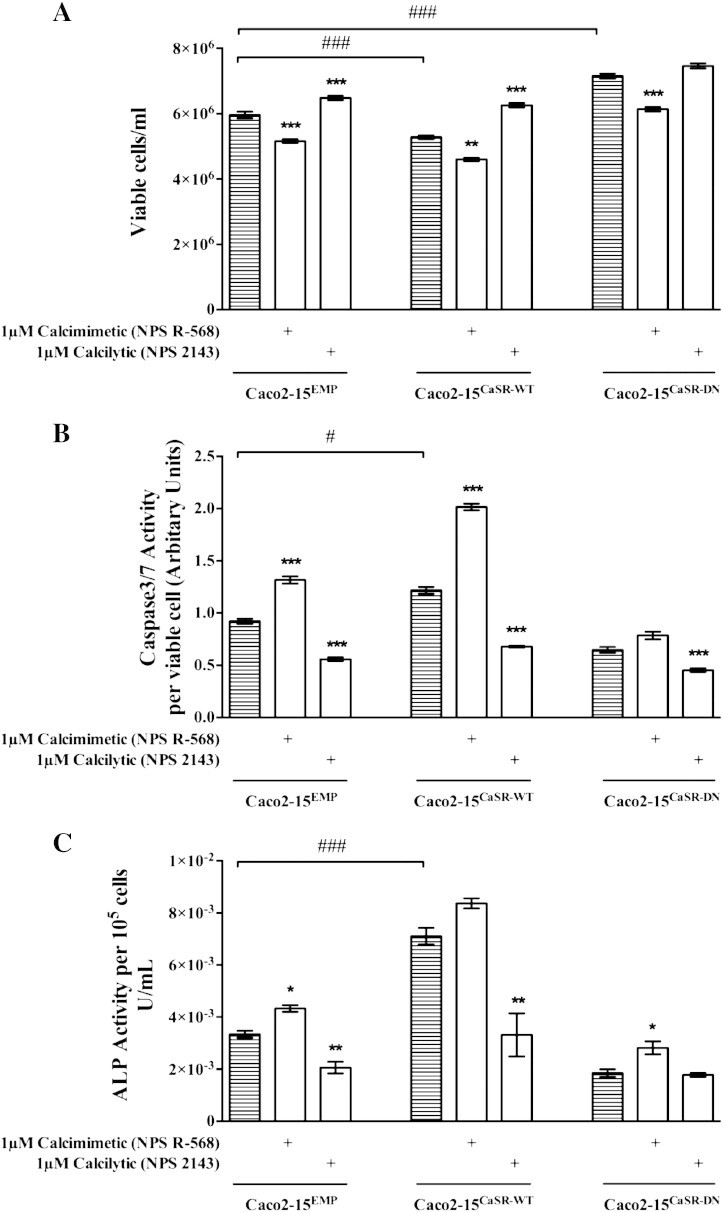
Impact of a CaSR activator or inhibitor on cell viability, differentiation, and apoptosis in stably transfected Caco2-15 cells. (A) Proliferation (n = 4), (B) apoptosis (n = 4) and (C) differentiation (n = 3) were measured in Caco2-15^CaSR-WT^, Caco2-15^CaSR-DN^ or the Caco2-15^EMP^ cells. Bars represent means ± SEM. Statistical significance was determined using ANOVA followed by Tukey's post-test. Asterisks above bars indicate significant differences within groups compared with the respective vehicle control (striped bars), whereas hash tags indicate significant differences between groups compared with Caco2-15^EMP^ cells. *p < 0.01, **p < 0.05, ***p < 0.001, ^#^p < 0.01 and ^###^p < 0.001.

Proliferation decreased significantly in the Caco2-15^CaSR-WT^ cells and in cells treated with the positive allosteric modulator NPS R-568 (Fig. 5A). These cells were more differentiated (as assessed by ALP activity, Fig. 5B) and had significantly increased apoptotic potential (Fig. 5C) compared with the Caco2-15^EMP^ cells. Furthermore, Caco2-15^CaSR-DN^ as well as cells treated with the calcilytic showed significantly decreased sensitivity to Ca^2 +^ as seen by increased cell number and decreased ALP and Caspase3/7 activity (Fig. 5).

### 3.6 Over-expression of CaSR inhibits proliferation and promotes apoptosis and differentiation in HT29 cells

To verify further the involvement of CaSR in inhibiting proliferation and inducing apoptosis we treated the stably transfected HT29 cells with either the calcimimetic NPS R-568, or the calcilytic NPS 2143 for 48 h.

**Fig. 6:**
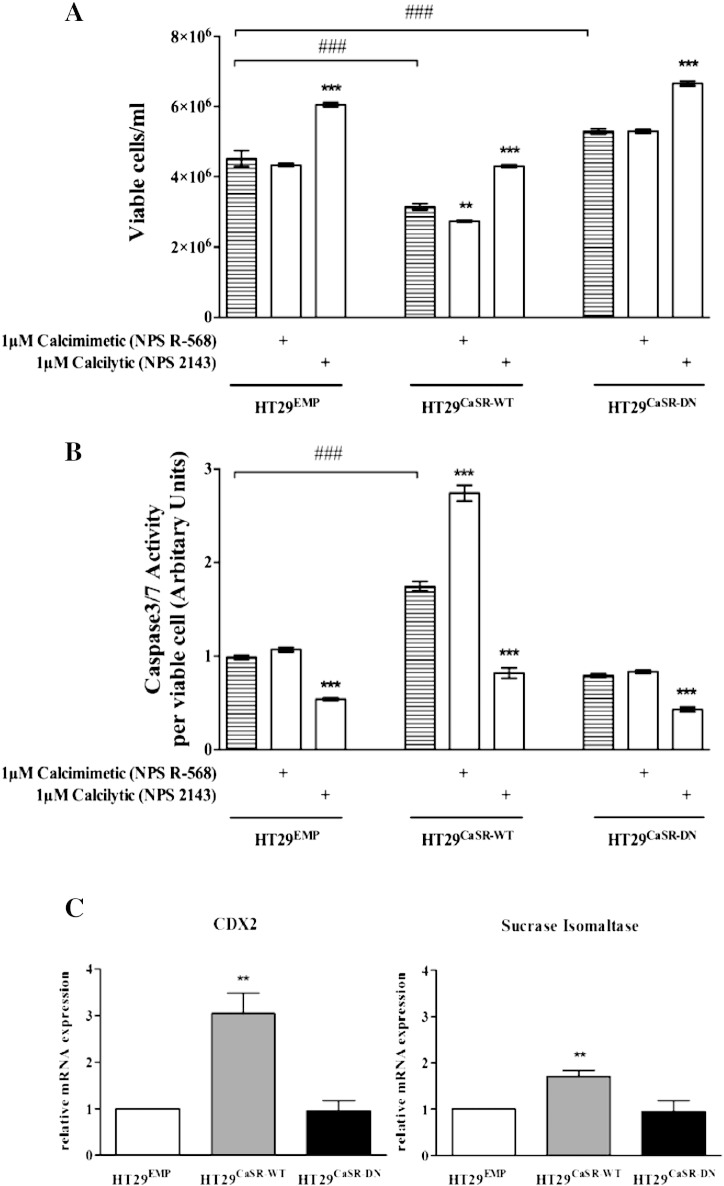
Impact of the CaSR on cell viability, apoptosis and differentiation in stably transfected HT29 cells. (A) Proliferation (n = 4) and (B) apoptosis (n = 3) were measured in HT29^CaSR-WT^, HT29^CaSR-DN^ or the HT29^EMP^ cells. (C) Gene expression of intestinal differentiation markers, SI and CDX2 was measured by qRT-PCR (n = 3). Bars represent means ± SEM. Statistical significance was determined using ANOVA followed by Tukey's post-test. Asterisks above bars indicate significant differences within groups compared with the respective vehicle control (striped bar), whereas hash tags indicate significant differences between groups compared with HT29^EMPTY^ cells. **p < 0.05, ***p < 0.001 and ^###^p < 0.001.

The positive allosteric modulator, NPS R-568 significantly decreased cellular proliferation (assessed by viable cell count, Fig. 6A) and increased apoptosis (assessed by Caspase3/7 activity, Fig. 6B) in cells overexpressing the CaSR (HT29^CaSR-WT^) compared with the HT29^EMP^ cells. Furthermore, in comparison to HT29^EMP^ cells, HT29^CaSR-DN^ cells as well as the cells treated with the calcilytic, NPS 2143 were less sensitive to Ca^2 +^ as seen by an increase in cell number, and decrease in Caspase3/7 activity (Fig. 6).

Consistent with previous studies, HT29 cells exhibited extremely low ALP activity in culture [28]. Therefore, we tested the impact of the CaSR on differentiation by evaluating the mRNA expression of the intestinal differentiation marker, sucrase isomaltase (SI) and caudal type homeobox 2 (CDX2) in the stably transfected HT29 cells. We saw a significant upregulation of both markers in HT29^CaSR-WT^ cells compared with HT29^EMP^ cells, whereas no difference was observed in HT29^CaSR-DN^ cells (Fig. 6C).

## 4 Discussion

Human and animal studies have shown that Ca^2 +^ prevents crypt hyperproliferation, suppresses dysplasia and protects the colon from malignant transformation [5,30]. The calcium-sensing receptor (CaSR) has been proposed as a potential mediator of these effects [6,31]. However, evidence for a causal relation between CaSR and colorectal tumorigenesis is still missing.

In the present study we show that CaSR expression negatively correlates with proliferation markers, but positively correlates with differentiation and apoptosis markers in samples from human colorectal adenocarcinomas. We demonstrate that knocking-down the CaSR indeed confers growth advantage to colonocytes *in vivo*. Finally, we produce evidence that restoration of CaSR function markedly suppresses the tumorigenic phenotype of CRC cells *in vitro* increasing their apoptotic and differentiation potential. Our data indicate that the CaSR is a colorectal tumor suppressor gene, which can be therapeutically targeted for chemoprevention of CRC. Moreover, we show for the first time, that loss-of-function mutations of the CaSR might be a risk factor for CRC.

Colonic CaSR expression is detected in normal mucosa, early adenomas and polyps; it is already decreased in advanced adenomas, and is almost undetectable in late stage, poorly differentiated tumors [20–22]. In the present study, we observed positive correlation between expression of the CaSR and expression of the differentiation marker SI and the pro-apoptotic marker BAX. SI is an independent marker of prognosis and survival in CRC patients [32,33]. Similarly, decreased expression of BAX predicts poor outcomes in CRC [34,35]. We show that both of these factors are increased in cells expressing functional CaSR supporting the hypothesis that downregulation of the CaSR is coupled to tumor dedifferentiation in CRC. CaSR expression levels correlated negatively with the DNA replication signature. This DNA replication signature, consisting of early replication genes, if upregulated during colorectal tumorigenesis, predicts poor prognosis in CRC [27].

It is already known that in intestine-specific CaSR knockout mice, the crypt architecture is disturbed and intestinal barrier function is impaired [17,36]. In the present study we observed significant upregulation in markers of proliferation, and a significant downregulation of markers of differentiation and apoptosis in DKO (*CaSR*^−/−^/*PTH*^−/−^) mice compared with control (*CaSR*^+/+^/*PTH*^−/−^) mice. Strikingly, lack of CaSR caused significant dysregulation of pre-neoplastic genes in the apparently normal colon samples from DKO mice. These mice spontaneously develop aberrant crypt foci and have increased susceptibility to dextran sulfate sodium-induced colitis [37]. In our study, the colonic tissue contained not only the epithelial layer, but also the stroma and muscularis. However, based on the data from Rey et al. [17] and Cheng et al. [36] using the intestine specific CaSR knock-out mouse as well as the CaSR/PTH double knock out model used by MacLeod [37] it appears that the CaSR in the epithelial cells is mainly responsible for these effects. Taken together, these data demonstrate that the CaSR is critical for maintaining normal intestinal cell turnover.

Intestinal epithelial cells are sensitive to extracellular calcium. In normal colonic epithelium, high calcium concentrations inhibit proliferation [5]. Epithelial cells at the bottom of the crypt proliferate optimally in the presence of low calcium concentrations (0.05–0.1 mM). As these cells begin to move up towards the crypt apex (where the calcium concentration reaches 0.8–2.2 mM), proliferation is turned off and the cells begin to differentiate [6]. During tumor progression, colon cancer cells can lose their sensitivity to the anti-proliferative effects of Ca^2 +^ [38], the causes for which are multifactorial and not completely understood. It has been reported that several calcium-activated, metastasis-promoting proteins (for e.g. members of the S100 protein family) become overexpressed during tumor progression [39]. Additionally, previous studies have shown that cells lacking the CaSR have an aggressive, highly malignant phenotype [21,40,41]. In the present study, we evaluated whether reintroducing the CaSR could restore sensitivity to the anti-proliferative effects of calcium. Therefore, we investigated the impact of the wild-type CaSR and a dominant negative mutant CaSR (R185Q) on the tumorigenic potential of two well characterized-CRC cell lines: the more differentiated Caco2-15 and the highly tumorigenic HT29 [26,28,29]. Caco2-15 cells, derived from a well-differentiated adenocarcinoma express detectable levels of endogenous CaSR. These cells differentiate spontaneously in culture and are sensitive to the antiproliferative, pro-differentiating effects of calcium [4]. HT29 is a highly malignant, adenocarcinoma-derived cell line with negligible levels of endogenous CaSR expression making these cells a good model to assess whether ectopic CaSR expression and function would restore their sensitivity to the anti-tumorigenic effects of Ca^2 +^. Our data shows that in Caco2-15 cells CaSR activation confers a differentiation boost; however in HT29 cells this effect is not as robust. In HT29 cells the main role of the CaSR seems to be stimulation of apoptosis, whereas in Caco2-15 cells the pro-differentiating effects seem to prevail.

We further confirmed the role of CaSR in mediating the anti-proliferative, pro-differentiating and pro-apoptotic effects of Ca^2 +^ by treating cells with the specific allosteric modulators of the CaSR: NPS R-568 or NPS 2143. Treatment with NPS R-568 enhanced the effect of Ca^2 +^, the primary CaSR agonist, in the cell lines expressing endogenous or exogenous wild-type CaSR suggesting that cells expressing functional CaSR could be targeted with synthetic positive allosteric modulators, as adjuvant treatment supporting traditional chemotherapy. Singh and colleagues have shown that in the presence of the CaSR, 5-Fluorouracil (drug of choice in CRC treatment) is more effective *in vitro*. Thus, specifically modified CaSR-pharmacochaperones that are ineffectively absorbed in the small intestine, but active in the colon, could be used to be highly effective locally without significantly affecting systemic Ca^2 +^ homeostasis.

**Fig. 7:**
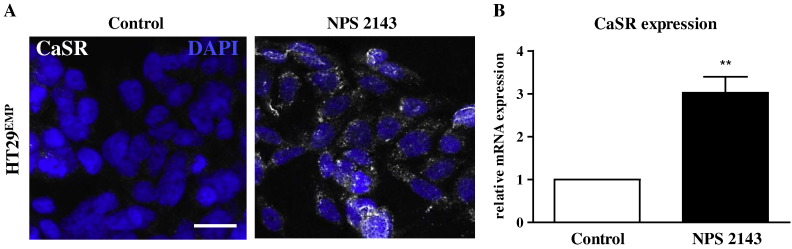
CaSR expression in HT29^EMP^ cells treated with NPS 2143. Expression of CaSR protein determined by immunofluorescence (A) and mRNA determined by qRT-PCR (B) in cells cultured in 1.8 mM calcium and treated with 1 μM NPS 2143. Vehicle-treated cells were used as control. White bar represents 50 μm. Asterisks above bars indicate significant difference compared with vehicle control. **p < 0.05.

The calcilytic, NPS 2143 enhanced proliferation, and inhibited apoptosis even in HT29^EMP^ cells which have negligible endogenous expression of the CaSR. These unexpected results suggest that the calcilytic must have additional effects besides modulating CaSR activity. Interestingly, 1 μM NPS 2143 upregulated endogenous CaSR expression in HT29 cells at both mRNA and protein levels (Fig. 7). Whether the effects seen in the functional data are due to the calcilytic targeting the newly synthesized endogenous CaSR, or are off-target effects (or both) needs further clarification.

In a recent case–control study, Dong et al. demonstrated a significant association between four *CaSR* gene variants, and the risk of development of carcinomas in the proximal colon [42]. The generation of transfectants over-expressing the dominant negative CaSR mutant R185Q allowed us to study the potential roles of the inactivating CaSR mutants in colorectal tumorigenesis. The R185Q mutant was previously identified in patients with Familial Hypocalciuric Hypercalcemia (FHH) [43] and neonatal severe hyperparathyroidism (NSHPT) [44]. A case report has previously shown that a patient with NSHPT bearing an R185Q mutation was successfully treated with cinacalcet, an FDA approved positive allosteric modulator of CaSR [45].

HT29^CaSR-DN^ and Caco2-15^CaSR-DN^ cells expressed the receptor at the cell surface [46], however they were resistant to the tumor protective actions of Ca^2 +^ consistent with previous findings that the residue R185 lies in close proximity to one of the proposed Ca^2 +^ binding sites [47], and that dimerization of mutant R185Q with wild-type CaSR reduces functionality of the latter [46]. In our experiments with Caco2-15 cells transfected with the CaSR-DN mutant, the treatment with NPS R-568 corrected the effect of the mutation and was able to reduce the malignant phenotype by increasing differentiation and reducing proliferation. Taken together, these results point towards a novel CRC risk group: patients with loss-of-function CaSR mutations, and suggest a potential role for CaSR modulators in treatment of disorders besides those of the parathyroid. Our subsequent goal is to evaluate whether patients with FHH and NSHPT who harbor inactivating mutations of the CaSR, indeed have increased CRC risk.

Our work provides evidence based on human patient data as well as mechanistic data from cell lines and animal models that the CaSR mediates the anti-neoplastic effects of calcium in the colon and protects the colonic epithelial cell from malignant transformation. We found that the stable over-expression of the functional CaSR affected several hallmarks of cancer cells: reduced growth, increased differentiation and induced apoptosis. We show for the first time that inactivating CaSR mutations could be a risk factor for CRC. Manipulation of CaSR expression and chemical modulation of CaSR function in cell lines demonstrates that the CaSR is indeed a functionally relevant target so that strategies aiming at either inducing CaSR expression or positively modulating its function are predicted to reduce cancer incidence. These data provide a rationale for suitably designed prospective trials using explicitly designed synthetic modulators that target the CaSR in colorectal cancer patients.

The following is the supplementary data related to this article.

Supplementary Table S1 Details of primers used in the study.

## Transparency documents

Transparency documents.

## Figures and Tables

**Table 1:** Clinicopathological characteristics of the patient cohort.

Patient characteristics	
Number	54
Gender	
Male	28
Female	26
Age (Mean ± SD)	68.7 ± 12.2
Tumor grading	
Grade 1	1
Grade 2	51
Grade 3	1
Unknown	1
Tumor staging	
Stage 1	11
Stage 2	17
Stage 3	25
Stage 4	1
Lymph node infiltration	
0	27
1	13
2	11
3	1
Unknown	2
Site of primary tumor	
Cecum/ascending/transverse	22
Descending/sigmoid	19
Rectum	13

**Table 2:** Correlation between expression of the CaSR and markers of neoplastic transformation in colorectal tumors. Spearman correlation coefficient (SCC) and p-value are shown.

	Function	SCC	p value
CDC6	DNA replication	− 0.502	< 0.001
CDT1	DNA replication	− 0.401	< 0.001
MCM2	DNA replication	− 0.369	< 0.001
MCM4	DNA replication	− 0.341	< 0.001
MCM6	DNA replication	− 0.481	< 0.001
MCM7	DNA replication	− 0.437	< 0.001
CDC45	DNA replication	− 0.500	< 0.001
SI	Differentiation	0.540	< 0.001
BAX	Apoptosis	0.310	< 0.001

**Table 3:** Impact of stable transfectants on doubling times in HT29 and Caco2-15 cells. Statistical significance was determined by ANOVA followed by Tukey's post-test. p values indicate significant changes within groups (compared with EMP cells).

	HT29		Caco2-15	
	Doubling time (h)	p value	Doubling time (h)	p value
Empty	13.0		17.4	
CaSR-WT	14.0	< 0.001	20.9	< 0.001
CaSR-DN	12.2	< 0.001	16.2	< 0.001
