# Supplementary material for: The calcium-sensing receptor: A promising target for prevention of colorectal cancer
Source: Biochim Biophys Acta. 2015 Sep;1853(9):2158–67. doi: 10.1016/j.bbamcr.2015.02.011 (PMC4549785; doi:10.1016/j.bbamcr.2015.02.011)
Supplement: Supplementary Table S1 — Details of primers used in the study. [file mmc1.docx]

|  | Forward | Reverse | Amplicon size |
| --- | --- | --- | --- |
| HUMAN |  |  |  |
| CaSR | GCCAAGAAGGGAGAAAGAC | CACACTCAAAGCAGCAGG | 154 |
| CDT1 | GGAGAAGCTCACCACTGCTC | CTTCTCCATCCTGGGTGAAA | 70 |
| MCM4 | TTGAAGCCATTGATGTGGAA | GGCACTCATCCCCGTAGTAA | 119 |
| MCM6 | AACCAGCAACTTTCCACCAC | ATCCTTGGCAAGAGGGATCT | 114 |
| MCM7 | CGGTGCTGGTAGAAGGAGAG | AAACCCTGTACCACCTGTCG | 115 |
| CDC45 | GCAGGTGAAGCAGAAGTTCC | TGTGCTTGAACCCAAAATGA | 140 |
| CDX2 | AGGGGGTGGTTATTGGACTC | CATTCAGCCCAGAGAAGCTC | 92 |
| SI | ACCGTTAAACATTCCAACCAC | GCCAAGAATCCCAAATGACTC | 131 |
| BAX | TGGAGCTGCAGAGGATGATTG | GAAAACATGTCAGCTGCCACTC | 82 |
|  |  |  |  |
| MOUSE |  |  |  |
| CaSR | GAGGCCTGGCAGGTCCTGAA | TGATGGAGTAGTTCCCCACC | 109 |
| Orc1 | CCTTTCAACAGATATACAGTCAGCA | GGAGGAGGTCATTTCTACTGG | 147 |
| Cdc6 | ACTCTCCGAATGTAAATCAC | CGAAATGACTTGGGATATGT | 79 |
| Mcm2 | GACCCTTCACTGACCTTCTC | CAGCATCTCATCCTGAACTG | 114 |
| Mcm5 | CTTCACTACCCAGGAGGAC | TGCTCTGGATATTTCTGTTTGG | 132 |
| Mcm6 | TGTTTCCAAGCCCTCCCT | ACTCGTCCTCCTCTTCTTCC | 101 |
| Cdc45 | ACGGTTAGAAGAGGAGATAGTG | GCCAGGTCAAACATCACCA | 144 |
| Si | ACAGCAAGCCGAAAGAATCC | TTCACCATCATCCCAGAAGAG | 90 |
| Bax | TGGAGCTGCAGAGGATGATTG | GAAAACATGTCAGCTGCCACTC | 82 |
| Eef1B2 | AGAGCTACATTGAGGGGTACG | GACTTGATGTGATTATACCAACGTAG | 118 |
| β-actin | TCCTAGCACCATGAAGATCA | CCACCGATCCACACAGAGTA | 66 |

**Supplementary Table S1.** Details of primers used in the study.
